# Hematochezia and colitis in a new diagnosis of anorexia nervosa in an 11-year-old boy: a case report

**DOI:** 10.1186/s40337-026-01737-0

**Published:** 2026-08-10

**Authors:** Colbey Ricklefs, Elizaveta Ignatova, Sara M. Buckelew

**Affiliations:** 1 Division of Adolescent and Young Adult Medicine, Department of Pediatrics, University of California, San Francisco, Box 0110, 550 16th St, San Francisco, CA 94143 USA; 2 Center for AIDS Prevention Studies, Division of Prevention Science, Department of Medicine, University of California, San Francisco, 550 16th St, Box 0110, San Francisco, CA 94143 USA

**Keywords:** Anorexia nervosa, Eating disorders, Malnutrition, Inpatient treatment, Hematochezia, Colitis, Inflammatory bowel diseases, Gastrointestinal complications

## Abstract

**Background:**

To our knowledge, this is one of the first case reports describing hematochezia and acute colitis as a consequence of refeeding in the setting of severe malnutrition. The differential diagnosis was broad and included ischemic (for example, possible reperfusion-like injury following refeeding), infectious, and autoimmune/inflammatory (for example, inflammatory bowel disease) etiologies. This presentation is rare, particularly in early adolescents and boys.

**Case presentation:**

This case report describes an 11-year-old boy who intentionally restricted his food intake for six months, resulting in a 17.1-kg weight loss (from 50.9 kg to 33.8 kg), and who presented after 72 h without oral intake. Shortly after initiating enteral nutrition via nasogastric tube, the patient developed significant hematochezia associated with a fever, tachycardia, and a marked elevation in C-reactive protein and fecal calprotectin. A computed tomography of the abdomen was concerning for diffuse ischemic colitis of unclear etiology, leading to a diagnostic laparoscopy, which demonstrated an edematous, acutely inflamed colon with no areas of ischemia.

**Conclusions:**

The present case highlights an unusual gastrointestinal complication of severe malnutrition and the refeeding process.

## Background

An estimated 90% of patients with anorexia nervosa (AN) report gastrointestinal concerns [1]. Patients with eating disorders commonly present with gastrointestinal complications of malnutrition, including nausea, vomiting, bloating, gastroparesis, and constipation [1]. Gastrointestinal complications secondary to severe malnutrition can affect any segment of the gastrointestinal tract and may result from malnutrition itself, purging behaviors, or the refeeding process [2].

Structural gastrointestinal issues in patients with eating disorders are frequently described in the literature, including gastric dilation, superior mesenteric artery syndrome, and rectal prolapse [3, 4]. There have been rare case reports of bowel ischemia or necrosis in patients with eating disorders, but these have been in adult female patients often after many months to years of severe malnutrition [5–11]. Only three cases have reported an association between AN and pneumatosis intestinalis [5, 12, 13], which can be a life-threatening sign of severe necrosis or perforation [14]. Proposed pathophysiologic mechanisms suggest that severe malnutrition may lead to intestinal atrophy and changes in the intestinal mucosa, but this evidence is often limited to areas with infectious outbreaks or in resource-limited settings, which may not be generalizable to patients with eating disorders [15].

A potential organic cause of malnutrition or weight loss may be inflammatory bowel disease (IBD). The clinical manifestations of IBD are diverse, ranging from bloody diarrhea, vague abdominal pain, fever, weight loss, or growth restriction [16]. According to the Revised Porto Criteria [16], the diagnostic standard for pediatric IBD, the standard workup requires esophagogastroduodenoscopy and ileocolonoscopy with biopsies from all segments, in addition to small bowel imaging via magnetic resonance enterography or wireless capsule endoscopy. These findings can support a diagnosis of ulcerative colitis, Crohn’s disease, IBD-unclassified (IBD-U), or no IBD. Histologic evidence of chronic colonic mucosal inflammation is a cornerstone of the diagnosis of all IBD subtypes. Fecal calprotectin is extremely sensitive in detecting mucosal inflammation and is superior to any serum biomarker, though serological markers such as ASCA or pANCA can help differentiate atypical cases [16].

Due to the overlap in symptoms between AN and IBD, including weight loss, abdominal pain, and altered bowel habits, these comorbid conditions can complicate or delay the diagnosis or treatment of each other [17]. There is evidence that pro-inflammatory cytokines, like tumor necrosis factor-alpha and interleukin-6, play a role in both AN and IBD [18, 19]. Furthermore, loss of appetite in IBD may positively reinforce restrictive eating behaviors in comorbid AN [17]. However, less is known about the potential shared mechanisms or underlying pathology that link the two diagnoses. A population-based study conducted over two decades in Denmark found that a diagnosis of AN was associated with an increased risk of developing IBD; however, IBD was not associated with an increased risk of developing AN [20]. A similar population-based study in Sweden identified a bidirectional association between AN and Crohn’s disease in women, but there was not a bidirectional pattern in men [21]. A systematic review of IBD and eating disorders yielded 219 cases with only 3 reported on male patients [22].

This case report describes an 11-year-old boy with severe AN who developed acute colitis and hematochezia early during refeeding, accompanied by elevated C-reactive protein and fecal calprotectin levels.

## Case presentation

An 11-year-old Latino boy presented to the emergency department after 72 h of refusal of all forms of oral intake. History was obtained from his parents, who reported that approximately six months prior, a peer called him fat at school. His parents noticed that he began sucking in his stomach without explaining why and started skipping breakfast but continued eating dinner. He later markedly restricted his nutritional intake, which ultimately culminated in three days without any oral intake.

He had no significant past medical or surgical history, no allergies, and took no medications. He lived with his parents and younger sibling in California’s Central Valley. There was no family history of IBD or celiac disease. There was a history of diverticulitis in the patient’s paternal great-grandmother and paternal great-uncle. There was no history of familial polyposis syndromes, thrombophilia, or platelet or bleeding disorders.

*Hospital Day 0*

Upon presentation, the patient was 33.8 kg (22nd percentile), down from 50.9 kg (93rd percentile) six months prior per medical records. His body mass index upon presentation was 18.0 kg/m^2^ (57th percentile, or Z = 0.17), a sharp decline from 23.6 kg/m^2^ (94.9th percentile, Z = 1.63) just two months prior. He presented with hypoglycemia (64 mg/dL) and bradycardia (40 beats per minute) and was admitted to a children’s hospital on the Eating Disorder Service for medical stabilization. Further history revealed that the patient reported “not wanting to get fat,” and that he was “aware he could die if he stops eating, but he would prefer not to be fat.” He began restricting by eating only fruits and vegetables for several weeks prior to presentation, avoiding all grains, dairy products, and meat, until 72 h prior when he refused to eat at all. On admission, his hemoglobin (Hgb) was 12.0 g/dL and his erythrocyte sedimentation rate (ESR) was 12 mm/hr, both within the normal range (see Table 1).

*Hospital Day 1*

The patient refused all oral intake, including nutritional supplements for food not eaten. A nasogastric tube was placed, but placement was difficult and required physical and chemical restraints including multiple doses of olanzapine (7.5 mg total). Nasogastric tube feeds were completed overnight to supplement the lost nutrition from the daytime.

*Hospital Day 2*

The patient was febrile to 38.5 °C in the morning. The patient developed frequent watery diarrhea after initiation of enteral feeds. His heart rate increased throughout the day. Point-of-care glucoses were within normal range during feeds.

*Hospital Day 3*

In the morning, the patient was tachycardic to over 160 beats per minute. His parents reported that the patient had blood-streaked stool and described abdominal pain the night before, and that morning had frank bloody stool. Enteral feeding was stopped due to tachycardia and hematochezia. His hypoglycemia improved to 277 and 291 mg/dL. The pediatric gastroenterology team was consulted and recommended a comprehensive laboratory evaluation in addition to computed tomography (CT) of the abdomen. The recommended laboratory workup included complete blood count, comprehensive metabolic panel, magnesium, international normalized ratio, prothrombin time, and blood typing and crossmatching. They recommended a fecal calprotectin, inflammatory markers (CRP and ESR), a celiac screening through immunoglobulin A (IgA) and tissue transglutaminase IgA (tTG-IgA), and a screening for mitochondrial disorders through a lactate-to-pyruvate ratio. Initial results revealed an elevated Hgb (16.7 g/dL), lactate (2.6 mmol/L), and CRP (102.4 mg/L), with normal levels of ESR (25 mm/hr), IgA (18 mg/dL), and tTG-IgA (< 10.0 CU; see Table 1).

The patient had ongoing hematochezia totaling six stools with frank blood over 24 h, with increasing amounts of blood. He remained afebrile but was tachycardic. His physical examination was normal except for mild abdominal tenderness. The patient was made nothing by mouth and a CT of the abdomen was completed later in the day.

The abdominal CT demonstrated “pneumatosis intestinalis, mural thickening, mesenteric edema, and interloop fluid involving nearly the entire colon with sparing of a portion of transverse colon and also involvement of small bowel loops in the left upper quadrant with relative mucosal hypoenhancement of a segment of jejunum. Findings are concerning for diffuse ischemic colitis of unclear etiology. Mesenteric and venous vessels appear grossly patent. No evidence of bowel obstruction or frank pneumoperitoneum.”

Given these laboratory results coupled with the new findings of extensive pneumatosis concerning for ischemia, the pediatric surgery team was consulted to explore and potentially remove areas of ischemic bowel. Diagnostic laparoscopy demonstrated areas of edematous, acutely inflamed and thickened distal small bowel and colon, including mesentery. There were no areas of ischemia, and so further laparotomy for bowel resection was not conducted.

The pediatric gastroenterology team considered a broad differential diagnosis, including (a) arterial occlusion (embolic or thrombotic), neither of which was evident on imaging; (b) venous obstruction; (c) nonocclusive ischemia, including hypoperfusion or localized ischemia from small-vessel disease; and (d) inflammatory bowel disease.

Initial laboratory results began returning, including a negative celiac and mitochondrial disorders screening. Stool studies were negative for all bacterial (campylobacter, E. coli O157, ETEC LT/ST, salmonella, STEC stx1/stx2, shigella, and vibrio cholera), viral (adenovirus 40/41, norovirus GI/GII, rotavirus A), and parasitic (cryptosporidium, entamoeba histolytica, and giardia) etiologies. The C. difficile toxin gene PCR was not detected, and there were no parasites identified on direct or concentrated smear.

*Hospital Days 4–8*

Hgb dropped to 10.8 g/dL by Hospital Day 4. After transfer to the pediatric hospital medicine team, a peripherally inserted central catheter was placed. Total parenteral nutrition was initiated to provide bowel rest, which resulted in resolution of his hematochezia.

*Hospital Days 9–12*

A fecal calprotectin level was collected on Hospital Day 9, which later returned markedly elevated at 1650 µg/g (ref: ≤49 µg/g). Repeat lactate level was 0.9 mmol/L. A repeat CT of the abdomen on Hospital Day 11 demonstrated interval resolution of extensive pneumatosis intestinalis and bowel wall thickening.

On Hospital Day 12, the pediatric gastroenterology team conducted an endoscopy, noting erythematous, congested, eroded and granular mucosa in the stomach but otherwise normal findings. A flexible sigmoidoscopy revealed a normal-appearing sigmoid colon and rectum, along with a normal perianal examination. Biopsies were taken.

*Remainder of Hospitalization (Hospital Days 13–26)*

Final results of the biopsy of the duodenum, stomach, esophagus, and sigmoid colon showed no significant pathologic abnormality. The biopsy of the rectum contained colonic mucosa with rare Paneth cell metaplasia. The pathology report noted that this finding can be a feature of chronicity, but there were no other features of chronicity, including no crypt architecture. There were no granulomas identified. On Hospital Day 20, a repeat fecal calprotectin was 190 µg/g and repeat CRP was 3.7 mg/L; Hgb was stable at 9.2 g/dL (see Table 1). Given the improved laboratory values, the pediatric gastroenterology team did not plan for colonoscopy.

Our psychologists with eating disorder expertise assessed and diagnosed him with severe anorexia nervosa, restricting subtype. He met DSM-5 criteria due to his restriction of energy intake (72 h of no oral intake after months of intentional restriction), intense fear of gaining weight or becoming fat (statements like “not wanting to get fat”), and disturbance in his body weight or shape (sucking in his stomach) [23].

The patient had complete resolution of his bloody stools, though continued to have severe cognitive rigidity with all nutritional intake, including via nasogastric tube. After reaching medical stability, he was ultimately discharged on Hospital Day 26 with plans to transfer to an inpatient medical behavioral unit for ongoing support of his eating disorder.

**Table 1:** Selected Laboratory Results

	Units	Ref.	HD0	HD3	HD4	HD9	HD11	HD12	HD20	PD
Hgb	g/dL	11.6–15.5	12.0	16.7	10.8	--	--	8.9	9.2	10.9
Lac	mmol/L	*1.0-2.4*	--	2.6	0.9	0.9	1.3	--	--	--
CRP	mg/L	*0.1-1.0*	--	102.4	108.5	--	--	--	3.7	0.5
ESR	mm/hr	*2–34*	12	25	--	--	--	--	--	52
IgA	mg/dL	*53–204*	--	186	--	--	--	--	--	--
tTG-IgA	CU	*< 20.0*	--	< 10.0	--	--	--	--	--	--
FC	µg/g	*≤ 49*	--	--	--	1650	--	--	190	--

HD: Hospital Day. PD: post-discharge, what would have otherwise been HD28

Hgb: hemoglobin. Lac: lactate. CRP: C-reactive protein. ESR: erythrocyte sedimentation rate. IgA: immunoglobulin A. tTG-IgA: tissue transglutaminase IgA. FC: fecal calprotectin

## Discussion and conclusions

This case presents an 11-year-old boy with severe restriction of nutritional intake who developed hematochezia and acute colitis during the refeeding process. The differential diagnosis for his acute colitis was broad: ischemic (possible reperfusion-like injury following refeeding), infectious (although no infectious etiology was identified on initial laboratory testing), or autoimmune/inflammatory (IBD). Imaging demonstrated no evidence of a single-vessel arterial or venous occlusion; however, the overall presentation may represent a relative ischemia–reperfusion injury. To our knowledge, this is one of the few reported cases documenting an association between severe malnutrition or AN and acute colitis with hematochezia in the absence of ischemic colitis. Even rarer is this complication in an early adolescent, a male patient, or a patient whose disordered eating behaviors started only six months prior, and who presented with a body weight within the normal BMI range.

Chronic symptoms of IBD may include restriction of intake and weight loss, which may be misattributed to an eating disorder, especially in certain age groups or with overlapping symptoms. Biochemical markers (namely, fecal calprotectin) have been reported as helpful to distinguish between IBD and eating disorders [24].

Fecal calprotectin is a protein that binds to calcium and zinc and is derived from neutrophils. It is a marker of intestinal inflammation, which makes it a useful tool in the diagnosis of IBD [25]. While fecal calprotectin is not disease-specific, it has a high predictive value for IBD and can be elevated in nearly 99% of patients with active IBD [25]. A meta-analysis identified the pooled sensitivity of fecal calprotectin to be 0.93 (95% confidence interval [CI]: 0.85 to 0.97) and pooled specificity to be 0.96 (95% CI: 0.79 to 0.99) in adults [26]. However, its utility is significantly lower (*p* = 0.048) in children and adolescents, in whom the pooled sensitivity and specificity were 0.92 (95% CI: 0.84 to 0.96) and 0.76 (95% CI: 0.62 to 0.86), respectively [26]. It is important to note that other common inflammatory markers, namely the ESR, may be low in the setting of weight loss in AN [27].

Our patient presented with a single episode of acute colitis during the refeeding process. He had a markedly elevated fecal calprotectin and CRP, along with Paneth cell metaplasia on biopsy. While these are findings that may suggest IBD, there was no other evidence of IBD, including granulomas, extraintestinal manifestations, or skip lesions. While it is possible that his refeeding triggered a new diagnosis of IBD, it is more likely that the refeeding process led to a reperfusion injury. Curiously, our patient also had a marked improvement in fecal calprotectin without immunosuppressive or induction therapy for IBD.

Clinicians caring for patients with either eating disorders or IBD should include both conditions in the differential diagnosis. Fecal calprotectin can be a useful tool to differentiate between the conditions, though the specificity may be lower in children and adolescents. Additional research on the interplay between IBD and eating disorders is needed, particularly among early adolescents and especially among boys.

## Data Availability

No datasets were generated or analysed during the current study.

## Abbreviations

AN: Anorexia nervosa
CRP: C-reactive protein
CT: Computed tomography
ESR: Erythrocyte sedimentation rate
FC: Fecal calprotectin
HD: Hospital Day
Hgb: Hemoglobin
IBD: Inflammatory bowel disease
IBD-U: IBD-unclassified
IgA: Immunoglobulin A
Lac: Lactate
PD: Post-discharge
tTG-IgA: Tissue transglutaminase IgA
